# Development and validation of a sodium AnaLysis tool (*SALT*)

**DOI:** 10.1186/s12937-020-00555-7

**Published:** 2020-06-15

**Authors:** Marcia Cooper, Janis Randall Simpson, Rita Klutka

**Affiliations:** 1 Bureau of Nutritional Sciences, HPFB, Health Canada, Sir Frederick G. Banting Research Centre, 251 Sir Frederick Banting Driveway, Ottawa, Ontario K1A 0K9 Canada; 2 Family Relations and Applied Nutrition, University of Guelph, Guelph, Ontario Canada

**Keywords:** Sodium, Screener, Intakes, Validation, Food frequency, Tool

## Abstract

**Background:**

Sodium is an essential nutrient; however, excess dietary sodium is associated with increased blood pressure levels. The 2004 Canadian Community Health Survey – Nutrition (CCHS 2.2) concluded that most Canadians exceeded the Tolerable Upper Intake Level (UL) of 2300 mg/day. The 2015 CCHS indicated that Canadians were still consuming above the UL. To assess population sodium intakes, a Sodium AnaLysis Tool (*SALT*) was developed.

**Methods:**

We used data from CCHS 2.2 (2004) to group foods into types (e.g., popcorn, crackers) and general categories (e.g., snack foods) which formed the *SALT* questions. Portion sizes and sodium values were calculated for *SALT* questions. Over a one-month period, one hundred participants completed three, 24-h recalls (at beginning, middle, and end) and two *SALT* (*SALT*_1_ & *SALT*_2_) tools (at beginning and end). To assess both validity and reliability, statistical tests including Bland-Altman (B-A) plots, paired t-tests, differences between means, and correlations were conducted. The mean of the 3,24-h recalls (m24HR) was used for validation.

**Results:**

Validity testing between *SALT*_*2*_ and the m24HR yielded variable results. A B-A plot between *SALT*_*2*_ and m24HR depicted a small bias of 7 mg/day of sodium. The sodium intake for m24HR (2742 ± 980 mg/day) (mean ± standard deviation) versus *SALT*_*2*_ (2735 ± 1174 mg/day) was not significantly different (*p* = 0.960). Pearson’s correlation between methods, although significant (*p* = 0.02) was poor (r = 0.202; de-attenuated r = 0.400). There was a fair, significant agreement (κ = 0.236, *p* = 0.02) for the classification of sodium intake into two categories (above or below the UL). Test-retest reliability results were also variable. There was moderate, significant agreement (κ = 0.488, *p* = 0.001) for classification of sodium intake into two categories between *SALT*_*1*_ and *SALT*_*2*_, a significant correlation (Pearson’s r = 0.785, *p* < 0.001), and the B-A plot depicted good agreement. However, the values for sodium intake for *SALT*_1_ (3185 ± 1424) vs *SALT*_2_ (2735 ± 1174) were significantly different (*p* = 0.005).

**Conclusions:**

Results indicate that the *SALT* has the potential to be a valid and reliable tool for assessing dietary sodium intake of Canadian adult populations. Despite some classification issues, there may be some value in using the *SALT* to categorize sodium intakes. Further refinement of the *SALT* may be required.

## Background

Sodium, a nutrient found abundantly in nature, is necessary for human health and normal functioning [1]. Although some sodium is necessary to regulate body fluid and blood pressure and to keep muscles and nerves functioning properly, excess dietary sodium is associated with increased blood pressure. Recent data suggest that approximately 25% of Canadians aged 20 years and older have been diagnosed with high blood pressure [2]. In 2004, the Institute of Medicine (IOM) released recommendations on sodium with the Tolerable Upper Intake level (UL) for salt set at 5.8 g per day (or 2300 mg sodium). The Canadian Community Health Survey – Nutrition (CCHS 2.2) conducted in 2004 showed that most individuals were exceeding the IOM recommended UL of 2300 mg/day [1]. The mean usual intakes for sodium were 3345–4083 mg/day for males 19–70 years of age and 2587–2778 mg/day for females 19–70 years of age [3]. For this age group, more than 85% of men and 69% of women exceeded the UL for sodium [4]. The major contributors to dietary sodium intake in Canada are commercially prepared foods, including those from restaurants and food services establishments. The key food group contributors of sodium are breads (14%), processed meats (9%), and pasta dishes (6%) [5].

To support the development of population health initiatives to reduce excessive sodium consumption it is essential to be able to measure sodium intakes in the population, including the proportion with intakes above the recommended amounts. Nationwide determination of sodium consumption requires nutrient intake assessment methods that can be easily disseminated across the country. Accurate measurement of sodium intake is difficult due to the extensive distribution of sodium in foods, the widespread use of sodium compounds in food processing, and the extensive use of table salt [6]. There are a limited number of short questionnaires in the literature for classifying individual sodium intakes and salt use; these questionnaires have generally been cumbersome, not validated, not developed for a Canadian population, or not for surveillance purposes. Charlton et al. developed and validated a 42-item food frequency questionnaire (FFQ) to assess habitual salt intake using representative dietary data from three ethnic groups in South Africa. It was found that the questionnaire considerably underestimated the dietary intake of sodium in the study population, due mainly to the intake from salt added by individuals at the table [6]. Other questionnaires included a short dietary questionnaire on salt use and salt preferences of individuals in Finland [7] and a diet history questionnaire to assess sodium and potassium intake, validated in Japanese adolescent and young adult populations [8]. The NutritionQuest Sodium Screener [9] was developed by examining the top sources of sodium intake by US adults using two large data files, one from FFQs and one from 24-h recalls (NHANES 2007–2008), and then selecting food items within the top 80% of sodium consumption. Most recently, a Canadian web-based 23-question screening tool for sodium, called the Salt Calculator, was developed to allow individuals to assess the amount and sources of sodium in their diets [10].

Dietary assessment tools for sodium intake are essential for informing public health interventions for dietary sodium reduction, as they enable the identification of sources and amounts of sodium intake. There are occasions when dietary records or full-length FFQs are not practical and therefore screening tools are developed to assess just one or two nutrients or food groups [11]. Validation studies of screening tools are important to assess whether the questionnaire is measuring what it should measure or to assess the degree to which the questionnaire agrees with a ‘gold standard’ or other standard measures of diet.

The purpose of this research was to develop and validate a Sodium AnaLysis Tool (*SALT*) as a rapid surveillance tool to be used to assess and to potentially categorize sodium intakes in the Canadian adult population.

## Methods

### Sodium AnaLysis tool (*SALT*) development

#### *SALT* questions

A review of the Health Canada report ‘Baseline sodium levels in the Canadian diet - Focus on Sodium Food Sources’, was conducted to help determine foods to be included on the *SALT* [12]. All individual foods and recipes captured by CCHS 2.2 (2004) were grouped into similar food types (e.g., popcorn, potato chips, crackers) and then combined into general categories (e.g., snack foods) which formed the basis of the questions on the *SALT*. Foods contributing less than 50 mg per serving of sodium were excluded from the groupings (e.g., yogourt, milk, water, etc). Response frequencies for each participant considered consumption in the last four weeks and each question consisted of monthly, weekly or daily options.

#### Serving sizes

Serving sizes were determined by matching *SALT* foods to median serving sizes previously calculated for another FFQ developed using CCHS 2.2 (2004) data [13]. Subsequently, weighted serving sizes reflecting Canadian consumption patterns were calculated for each *SALT* question using male and female grams per capita from CCHS 2.2 (2004) and Canadian Diet History Questionnaire II (DHQ) serving sizes [13].

#### Pilot testing

Prior to finalizing the *SALT*, it was piloted in Ottawa, ON with 15 Canadian adults who had similar characteristics to the desired study population. After completion of the *SALT*, participants were asked questions related to their understanding of portion sizes, reduced-sodium foods and interpretation of specific food categories. After adjustments were made based on the pilot study, the finalized *SALT* included forty food-based questions with three additional items on discretionary salt intake [see Additional file 1].

#### Sodium values used for data analysis

Sodium values assigned to questions on the *SALT* were estimated based on a stepwise process.

In order to determine sodium values for each of the categories, CCHS 2.2 (2004) food and recipe codes were first reviewed and updated with Canadian Nutrient File (CNF) 2015 substitutions for deleted foods and finally sodium values were updated for all foods to reflect 2015 values [14]. Weighted sodium values were then calculated for each *SALT* question for males and females on grams of sodium per capita. Food consumption data from participants (24-h recalls and *SALT*) were collectsed in 2012–2013. This was combined with food composition data from CNF (2015) to generate sodium intakes for validation.

#### Reduced sodium values

A check box was included next to each question on the screener for participants to report the usual consumption of reduced or low sodium products within the food category. In order to assign values for low sodium food choices, a review of CCHS 2.2 (2004) foods and low sodium options present on the market (CNF [14], Nielsen market share data [15], Mintel GNPD - Global New Products Database [16], grocery store visits) was conducted; values relevant to foods on the Canadian market were substituted where appropriate. Weighted reduced or low sodium values for the *SALT* were calculated for each applicable *SALT* question based on grams per capita.

### Validity and reliability testing

#### Participants

Inclusion criteria for the validation and reliability study were that participants were 19 years of age or older and able to read English at a Grade 8 level [17]. The sample size for this study was calculated using a formula based on the Bland-Altman (B-A) Limits of Agreement [18]. We made assumptions based on the literature that the expected average difference of response between the *SALT* and the 24-h recalls would be 75 mg which was partially based on the expected difference between food diaries and a food frequency questionnaire [19]. Given some uncertainty in our estimates of the difference and standard deviation, a coefficient of variation level of 10% provided an acceptable margin of assurance that the estimated B-A Limits of Agreement would be reliable enough to validate the *SALT*. This corresponded to a sample size of 93 respondents to estimate the lower B-A limit for sodium. A sample size of 100 was estimated to be large enough to allow the limits of agreement to be estimated precisely [20].

We consecutively recruited 100 people, without oversampling, who gave us a full data set. A research assistant recruited the subjects on a rolling basis until all of the subjects were obtained.

Participants were recruited from a variety of locations within a 100 km radius of Guelph Ontario Canada from 2012 to 2013. The research assistants placed posters, used word of mouth and emails at various sites to recruit participants. Participants were not initially informed that the study focused specifically on sodium in order to avoid any potential bias or social desirability effects. Once the participants completed the study, they were informed on the true focus of this study – to validate a sodium-specific questionnaire. The study was approved by the Health Canada and the Public Health Agency of Canada’s (PHAC) Research Ethics Board (REB#2011–0027) and the University of Guelph Research Ethics Board (REB#13JA047). Informed written consent was obtained, and all participants received compensation of $25 Canadian as a token of appreciation for their participation.

#### Validity and reliability

To validate (relative validity) the tool, the results of the *SALT* were compared with the results of the mean of the sodium intake calculated from the three, 24-h dietary recalls (m24HR) that included dietary information for each subject for two weekdays and one weekend day. The second administration of the screener (*SALT*_*2*_) at the end of the ~ one month data collection period was used in the validation analysis as the tool asks about intake over the past month and reflects the data collected by the 3, 24-h recalls [21]. The use of multiple 24-h recalls that cover the same period of time as the FFQ is commonly employed in FFQ validation studies [20, 22].

Participants were asked to attend three separate interviews, each held at least one week apart and led by trained research assistants from the University of Guelph. The interviews were conducted in English. The *SALT* was administered prior to the 24-h recalls in order not to influence the participants’ responses when completing the screener [20]. During the first interview, participants completed the *SALT*, a 24-h recall and a short general questionnaire asking about demographic and eating out information. During the second interview, participants completed just a 24-h recall. At the third interview, the participants completed the *SALT* a second time (for test-retest reliability) and a 24-h recall.

In order to ensure that all participants received the same standardized 24-h recall, research assistants were trained in the multiple pass method [23] using food models from the CCHS 2.2 (2004) survey. Participants gave their responses orally while the research assistant(s) recorded those responses. Details that influence sodium composition such as added condiments, spreads, and cooking methods were recorded. Whenever possible, recipe ingredients, product brand names and grocery store or restaurant sources were obtained.

#### Data analysis

The frequency data from the *SALT* were converted to sodium intakes based on a representative composite value for each item on the screener. The 24-h recalls were processed by trained research assistants at Health Canada using the Nutrition Survey System, a program that included food descriptions from the Canadian Nutrient File [24] and a recipe file. Data from the 3, 24-h recalls were adjusted for usual intake using the Software for Intake Distribution Estimation (SIDE) program from Iowa State University [25]. Variance estimates (between and within participants) for the 24-h recall data were also calculated [26].

Data analyses were conducted using SPSS, version 26 (SPSS Statistics, IBM corporation, Armonk, NY, 2019). Descriptive statistics (mean ± standard deviation) were used for analysis of demographic and sodium use data. Spearman’s correlations tested associations between sodium intake and frequency of use of salt in cooking and at the table. The internal consistency of the *SALT* was determined using Cronbach’s alpha (a measure of internal consistency) [27].

The data (m24HR, *SALT*_*1*_*, SALT*_*2*_) were not normally distributed as assessed by Shapiro-Wilk’s test (*p* < 0.05 for all data sets); further data were positively skewed with skewness (standard error (SE) of 1.32 (0.24), 1.20 (0.24), and 0.75 (0.24) for m24HR, *SALT*_*1*_*, SALT*_*2,*_ respectively. Three outliers were detected that were more than 1.5 box-lengths from the edges of the box (interquartile range) in a boxplot [27]. Inspection of their values did not reveal them to be extreme (< 3 box-lengths) and they were kept in the analysis [25, 27].

A variety of statistical tests were employed to determine both the relative validity (*SALT*_2_ vs m24HR) and the test-retest reliability (*SALT*_1_ vs *SALT*_2_). These included B-A plots as they are the preferred method for depicting the agreement between two methods, particularly in the development of FFQs [20, 22, 28]. In addition to the B-A plots, paired samples t-tests were used to test whether there were statistically significant differences between the *SALT*_2_ and the m24HR (both unadjusted and SIDE-adjusted for usual intake) and between *SALT*_1_ and *SALT*_2_. As the data were positively skewed, square root transformations were applied to normalize the data before the t-tests were applied [29]; data were subsequently normal for *SALT*_1_ and *SALT*_2,_ but not for m24HR. Correlations between the *SALT*_2_ and the m24HR, and between *SALT*_1_ and *SALT*_2_, were conducted using Pearson’s correlation coefficient on log transformed data. The correlation between the *SALT*_2_ and the m24HR was conducted with and without de-attenuation for random error (using the variance estimates) from the 3, 24-h recalls [26]. For Pearson’s correlation, the strength of the association between the variable is considered very strong if the coefficient ranges from 0.8 to 1.0, moderate from 0.5 to 0.8, fair from 0.2 to 0.5 and very weak when less than 0.2 [30]. The validity of the *SALT* (*SALT*_2_ vs m24HR) and the test-retest reliability (*SALT*_1_ vs *SALT*_2_) to correctly classify sodium intakes into two categories (i.e., < 2300 mg/d vs ≥2300 mg/d), was assessed using the Cohen’s Kappa (κ) test [27, 31]. These categories were based on the UL for sodium as established by the IOM [1]. Landis and Koch [32] suggested that a κ of < 0.2 should be taken as representing ‘poor’ agreement, 0.21–0.40 as ‘fair’ agreement, 0.41–0.60 as ‘moderate’ agreement, 0.61–0.80 as ‘substantial’ agreement and 0.81–0.99 as ‘almost perfect’ agreement. A κ coefficient of 1 represents perfect agreement. Cross-classification analysis was performed to determine whether there was good agreement between the *SALT*_2_ vs m24HR, and to estimate the percentage of participants classified into the same or an adjacent quartile [33].

## Results

### Demographic and other characteristics

Data on demographics, frequency of eating out at fast food and sit-down restaurants and salt use are presented in Table 1 (*n* = 100). Briefly, most participants were female, young (19 to 30 years old), and born in Canada. There were no gender, nationality or age differences in sodium intakes. There was a positive significant, but fair, correlation between the frequency of eating out and sodium intake (Spearman’s rho = 0 .278, *p* = 0.006). There were no significant associations between sodium intake and frequency of use of salt either in cooking or at the table.

**Table 1:** Demographic and other characteristics of 100 participants from Southern Ontario

Variable	Percent of Sample (%)
Gender	
Male	18
Female	82
Country of Birth	
Canada	77
Other	23
Age Category (years)	
19–30	60
31–50	21
≥50	19
Frequency of Eating Out	
≥ 3 times/week	13
1–2 times/week	29
2–3 times/month	36
1 times/month	12
< 1 times/month	10
Salt Used for Cooking	
≥ 1 times/day	24
< 1 times/day	22
≤ 1 times/week	54
Salt Used at the Table	
≥ 1 times/day	6
< 1 times/day	15
≤ 1 times/week	79

Data for sodium intake for the m24HR and for the first (*SALT*_1_) and second (*SALT*_2_) administrations are shown in Tables 2 and 3. Mean sodium intake was above the UL (2300 mg/day) for the m24HR and for both *SALT* administrations. Further, more than 60% of participants had sodium intakes above the UL; fewer than 10% had sodium intakes less than 1500 mg/day.

**Table 2:** Relative validity of sodium intake estimated by the Sodium AnaLysis Tool and 3, 24-h recalls

Sodium Intake mg/day mean ± SD		Significance *SALT*_2_ vs m24HR	Mean Difference *SALT*_2_ vs m24HR mg/day		Pearson’s Correlation Coefficient *SALT*_2_ vs m24HR mg/day		
*SALT*_2_	m24HR	P value^a^	Mean^b^	%^c^	Crude r	Variance ratio^d^ (S_2_w/S_2_b^e^)	De-attenuated r_c_^f^
2735 ± 1174	2742 ± 980	0.96	−7	−0.3	0.202_g_	0.49	0.400

*SALT* Sodium AnaLysis Tool, *SALT*_1_ the first *SALT*, *SALT*_2_ the second *SALT*, m24HR the mean of the 3, 24-h recalls

*SD* standard deviation

^a^ paired t-test: t (99) = 0.05; Cohen’s d < 0.02

^b^ mean difference for *SALT*_2_ vs m24HR calculated as *SALT*_2_ - m24HR

^c^ % mean difference for *SALT*_2_ vs m24HR calculated as (*SALT*_2_ – m24HR)/m24HR) × 100

^d^ Variance ratio was calculated from 3, 24-h recalls

^e^ S_2_w/S_2_b within-person variation/between-person variation

^f^ de-attenuated r (r_c_) calculated as r_c_ = r_o_√[+S_2_w/S_2_b]/n, (r_o_ = observed correlation and n = number of replicates)

^g^*p* < 0.05

**Table 3:** Test-retest reliability of the Sodium AnaLysis Tool

Sodium Intake mg/day mean ± SD		Significance *SALT*_1_ vs *SALT*_2_	Mean Difference *SALT*_1_ vs *SALT*_2_ mg/day		Pearson’s Correlation Coefficient *SALT*_1_ vs *SALT*_2_ mg/day
*SALT*_*1*_	*SALT*_2_	P value^a^	Mean^b^	%^c^	R
3185 ± 1424	2725 ± 1174	0.005	− + 460	−17	0.785^d^

Sodium AnaLysis Tool (*SALT*), *SALT*_1_ the first *SALT*, *SALT*_2_ the second *SALT*

*SD* standard deviation

^a^ paired t-test: t (99) = 4.29; Cohen’s d = 0.45

^b^ mean difference for *SALT*_1_ vs *SALT*_2_ calculated as *SALT*_1_ - *SALT*_2_

^c^ % mean difference for *SALT*_1_ vs *SALT*_2_ calculated as (*SALT*_1_ – *SALT*_2_)/*SALT*_2_) × 100

^d^*p* = 0.001

### Validity

**Fig. 1:**
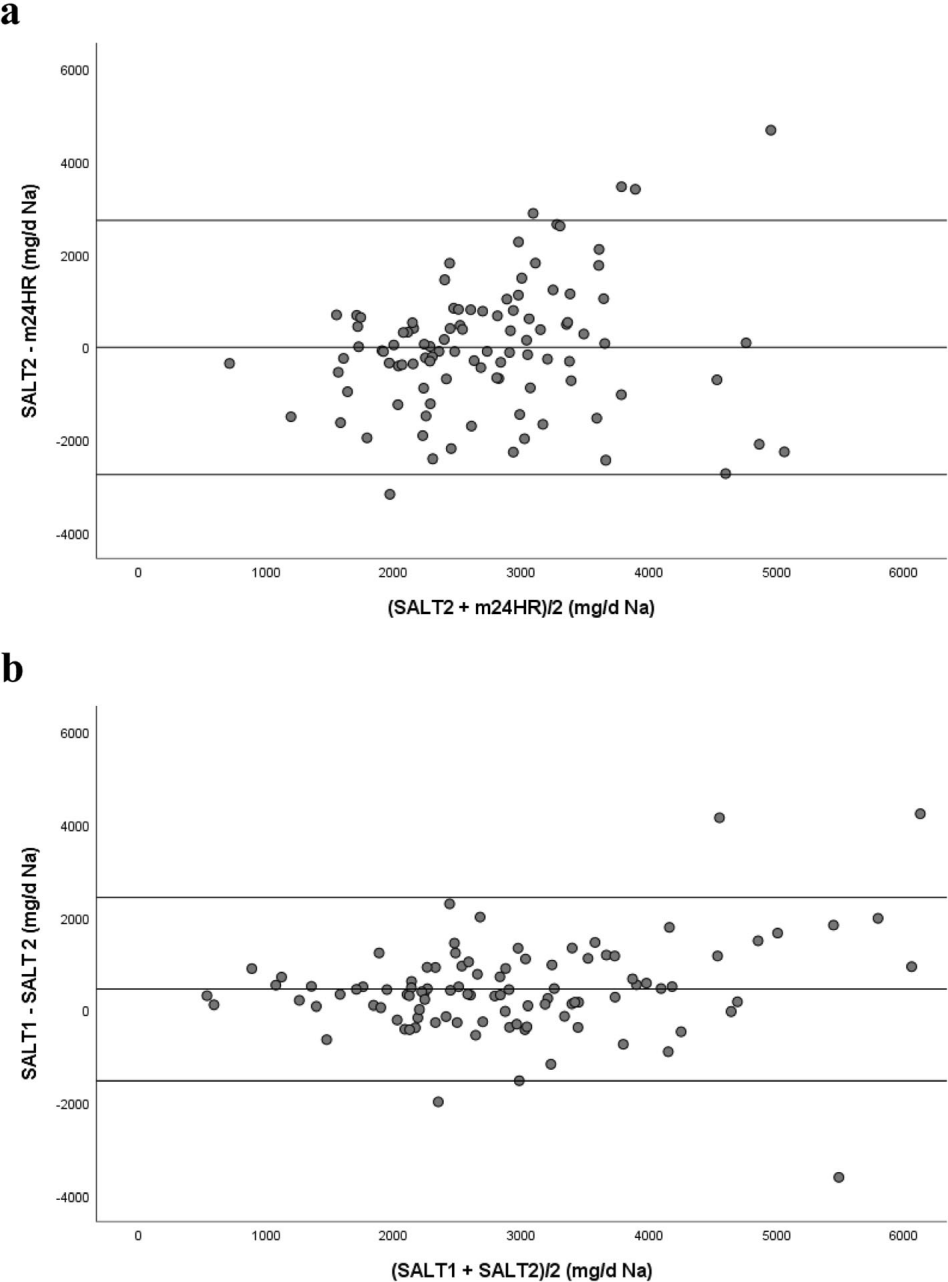
**a** Bland-Altman plot of the mean and difference of sodium (Na) intake from the mean of 3, 24- h recalls (m24HR) and the second administration of the Sodium AnaLysis Tool (*SALT*_*2*_)/ **b** Bland-Altman plot of the mean and difference of sodium (Na) intake from *SALT*_*1*_ and *SALT*_*2*_. m24HR = mean of 3, 24-h recalls; *SALT*_*1*_ = the first *SALT*; *SALT*_*2*_ = the second *SALT* The upper and lower lines represent the 95% confidence limits

In Fig. 1a, the agreement between the sodium intake estimated by the m24HR and *SALT*_2_ is depicted in a B-A plot. The *SALT*_*2*_ underestimated sodium intake by 7 ± 1161 mg/day. There was, however, a slight proportional bias for greater differences between the screener and the means of the 3, 24-h recalls with increasing sodium intake. The limits of agreement were wide given the large standard deviation of the difference. As expected, 95% of the data points fell within the limits of agreement.

Results of the data analysis for the paired samples t-test between the m24HR and the *SALT*_2_ were similar using both the original and the square root transformed data. Likewise, the results of the data analysis using the unadjusted and the adjusted (for usual intake) m24HR data were similar. Therefore, the results from the untransformed, unadjusted data are presented in Table 2 for the paired samples t-test. There was no statistically significant difference between the sodium intake from the m24HR and the *SALT*_2_. Further, there was a very small mean difference between the two methods (Table 2). As shown in Table 2, there was a significant, but fair, correlation between the m24HR and *SALT*_2_. Correction for de-attenuation of the data resulted in a somewhat larger *p* value; however, the correlation remained fair. Cohen’s Kappa (κ) was run to determine the agreement of the classification of sodium intake into binary categories (< 2300 m/day vs ≥ 2300 mg/day) between the m24HR and the *SALT*_*2.*_ There was significant (*p* = 0.02), but poor (κ = 0.236) agreement for 64% of categorizations (20% with sodium intakes < 2300 mg/day and 44% with sodium intakes ≥2300 mg/day). However, 17% had observed (m24HR) sodium intakes < 2300 mg/day vs predicted (*SALT*_2_) intakes of ≥2300 mg/day; 19% had observed sodium intakes ≥2300 vs predicted intakes of < 2300 mg/day.

#### Reliability of the SALT

The time interval between the two administrations of the *SALT* was 19 ± 5 days. The *SALT* demonstrated internal consistency with a Cronbach Alpha of 0.81. As shown in Fig. 1b, *SALT*_*1*_ overestimated sodium intake by 450 ± 1008 mg/day of sodium compared to *SALT*_*2*_*,* with no proportional bias. As shown in Table 3, the two administrations of the *SALT* were significantly correlated. There was a moderate and significant agreement based on Cohen’s Kappa for the classification of sodium intake from the first and second administrations of the *SALT* into two categories (κ = 0.488, *p* = 0.001). Seventy-seven percent of categorizations were in agreement; 21% were in agreement for sodium intake < 2300 mg/day and 56% were in agreement for sodium intake ≥2300 mg/day.

Results of the data analysis for the paired samples t-test between the first and second administrations of the *SALT* were similar using both the original and the transformed data. Therefore, the results from the untransformed data were used for the paired samples t-test and the correlation. As shown in Table 3, sodium intake from the first administration of the *SALT*_*1*_ was significantly higher than the intake from the second administration of the *SALT*_*2*_.

## Discussion

This study involved the development of a surveillance tool for the assessment of sodium intakes in a population of adults. Results of this study indicate that the *SALT* has the potential to be a valid and reliable tool for assessing dietary sodium intake based on the comparison with the 24-h recalls. The results were mixed with the B-A plot and the mean difference between methods suggesting validity; however, the correlation between the methods was fair (with a correction for de-attenuation). Our results are similar to those of Tangney et al. in their evaluation of the NutritionQuest Sodium Screener [21]. There is also the potential for using the *SALT* to assess the proportion of the population with sodium intakes above or below the UL although further work should be undertaken to address classification issues. Based on an assessment with 100 individuals, the *SALT* reliably categorized above and below this threshold 64% of the time.

The tool developed for this study provides a relatively quick dietary assessment surveillance tool to evaluate population sodium intakes and with some modifications there could be improved congruence to a 24-h recall. As is often typical of screeners, the *SALT* contained a short FFQ, without portion size questions, plus three behavioural questions on discretionary salt intake [34]. To the best of our knowledge, this is the first Canadian tool for dietary sodium intake assessment at the population level to undergo validity and reliability testing. Validation is a key component when determining whether a dietary assessment instrument is suitable for assessing intakes. Although the web-based Salt Calculator is not a validated tool, it has shown merit as a quick tool that identifies sources of sodium in the diet of individuals [10]. The *SALT* was validated against multiple 24-h recalls to evaluate the level of agreement against a ‘gold standard’ [20]. Typically, there are fewer measurement errors associated with dietary recalls compared with FFQs as they are less memory dependent and allow for accurate description of food and portion sizes using food models [23].

Agreement between the two methods may inherently be lower due to the nature of the recall. Sodium is a nutrient in which intra-individual variability in daily sodium intake can vary greatly (a difference of 897–1403 mg/day) [35]. Thus, this intra-individual variability can greatly influence the ability to have strong agreement and validity between dietary assessment methods. There was a mean difference of 7 mg of sodium between the mean of the 24-h recalls and *SALT*_2_ that was a non-significant difference indicating that as a population surveillance tool similar sodium levels could potentially be estimated. The points on the B-A plot were well-scattered, over and above zero, suggesting that there is no consistent bias between the SALT and the reference method of the 24-h recalls. The plot for our data is similar to data from the evaluation of the NutritionQuest Sodium Screener that also has wide limits of agreement [21].

A 2002 review of validation studies of FFQs noted that all FFQs should be validated with a sample of participants from a population in which the tool will routinely be employed [20]. It was suggested that FFQs be compared with results obtained from suitable reference methods (such as 24-h recall), and it be administered on multiple days over a similar period of assessment as the FFQ. A strength of the *SALT* validation is that it was administered twice and it was compared with the results of three 24-h dietary recalls administered during the same period.

FFQs have been suggested as the best method for estimating sodium intake as they can assess intake over an extended time period as compared to dietary recalls, while potentially dealing with issues of high day-to-day variability of sodium intake [36]. Three to 10 days of intake have been reported in the literature as needed to accurately measure usual intake of sodium [37]. Since we measured three days of intake via the 24-h recalls, the sodium values obtained in this study should reflect a reliable measure of usual intake.

The CCHS 2.2 (2004) mean usual intakes for sodium were 3345–4083 mg/day for males 19–70 years of age and 2587–2778 mg/day for females 19–70 years of age. Among respondents aged 19–70 years, more than 85% of men and 69% of women exceeded the UL for sodium [4]. Not surprisingly, the present study showed a mean sodium intake above the UL for both the *SALT* and the 24-h recall data. While the percentage (> 60%) above the UL was lower than that found in the CCHS 2.2 (2004) data, this lower value may be due partially to the 2010 Health Canada recommendations [38] which highlighted consumer education and set benchmark levels which could have led to some product reformulations.

A 2010 study of food packages suggested that lower-sodium products constitute about 4.5% of packaged foods in Canada [39]. Our study showed that overall, 32% of participants consumed lower sodium products once per month, 13% chose such a product weekly, while 4% consumed such products daily. Thus, our results reflect the reality that, as much as there has been push to reduce sodium intakes in the population, few people were regularly buying food advertised as reduced sodium foods on a regular basis [39]. The most consumed lower-sodium products were canned soups (11.5%), snacks (9.5%), gravies and condiments (9% each category), bread (8%), and canned vegetables (8%). The low intakes of sodium reduced products may have been partly due to the age of participants in our study who were predominantly (60%) between the ages of 19 and 30 and may not have been routinely seeking out reduced sodium products. Overall, there is a lack of data on the intakes of low-sodium products. Similar to the CCHS 2.2 (2004) data which reported that 30% of individuals older than 19 years never add salt at the table [4], the current study reported that 29% of individuals use salt at the at the table ‘never or less than once a month’. Therefore, there was still a propensity for some individuals in the study to salt their food at the table.

Results of consistency testing indicate that the *SALT* has high internal reliability. There was a significant positive correlation between *SALT* administrations. Further, test-retest reliability was demonstrated for assessing population sodium intakes based on classification into two categories, above and below the UL for sodium. Thus, there was good test-retest reliability for the *SALT* despite the mean intakes between the first and second administration being significantly different; however, the effect size was moderate. Since the tool asked about food consumption in the past four weeks and was administered on average almost three weeks apart, there was a possibility that the types of foods consumed or the frequencies of consumption were different. The value of an FFQ is reflected by the questions and the instructions provided to the respondent [20]. Despite pilot testing the *SALT* and getting feedback on ease of use of food categories and instructions, some participants could have classified the same foods under different categories between the iterations of the screener. For example, there are three vegetable and two soup categories and although there were some examples provided for most food categories there was the potential to check off the wrong category as participants moved through the screener. The screener could benefit from more examples under each category and more descriptive instructions. FFQs and screeners are developed to measure usual dietary intake by asking about consumption over an extended period of time. Although the screener asked the participants to think of what they consumed in the past month, there was potential for people to report on current consumption vs usual consumption [40].

Since the *SALT* was developed, the 2015 Canadian Community Health Survey (CCHS) data were collected, a national health survey asking Canadians ages 1 and older in every province about their self-reported eating habits and use of nutritional supplements, as well as other health factors [41]. In 2015, Canadians on average were still consuming above the UL for sodium. Since the sodium values for both the 24-h recall data and the *SALT* data were based on recent updated marketplace values, the validation of this tool is not impacted. However, it is recognized that consumption patterns and sodium values may have changed between 2004 and 2015 warranting a re-evaluation of the data used to create sodium values and serving sizes assigned to *SALT* categories.

Strengths of this research include an adequate sample size to determine validity and reliability of the tool, with research conducted over three seasons (excluding winter). Additionally, participants were from a large geographic area of Southern Ontario, thus contributing to some heterogeneity in the population although this should not have influenced the validity of the tool. The use of nationally representative Canadian population data to develop the food categories, portion sizes and sodium values associated with each of the categories reduced errors due to FFQ designs. Further, in order to get proper values for lower sodium foods, a detailed review of the marketplace was gathered along with CCHS 2.2 (2004).

There are limitations of this study that must be addressed. The majority of the participants were female; although this should not have influenced the validity of the tool, future work could focus on validating the *SALT* with more men. Most of the subjects were born in Canada; therefore studying the utility of the tool with individuals from different backgrounds would add to the generalizability of the *SALT*. Currently the categories within the *SALT* do not cover or provide examples of ethnic dishes which could be confusing for certain individuals who mostly consume foods not currently identified in the examples on the *SALT*. Thus, the *SALT* should be adapted to be more encompassing of ethnic foods. It was expected that the concordance between, the *SALT* and the m24HR would be stronger. The concordance between the m24HR and the *SALT* is moderate (64%), but is similar (59–70%) to that obtained recently in an evaluation of a Sodium Screener [21]. Similar to that research, the non-specific nature of the categories on the *SALT* could result to some discrepancy in alignment to the intakes from the 24-h recalls. With respect to total sodium intake, 67% of the subjects were correctly classified by the *SALT* into the same category (quartile) or the adjacent category as the m24HR, while 14% were grossly misclassified.

Finally, it is not surprising that the *SALT* validation demonstrated varied results based on the statistical tests. A review of the literature on methods used to determine the validity of dietary assessment tools suggests that using different statistical tests provides for insights and interpretation on different aspects of validity [42]. The multiple statistical tests used to test validity in the current study, provided a broader insight into the method chosen to assess sodium intakes in the population.

## Conclusions

In conclusion, this study has demonstrated that the Sodium AnaLysis Tool has the potential to be a valid and reliable instrument to assess sodium intakes of Canadian adult populations. The use of Canadian survey data has ensured the creation of a questionnaire that reflects typical Canadian consumption patterns. This work methodically established categories of foods that reflected consumption patterns and sodium contributors. Despite some classification issues, there may be some value for using the *SALT* to categorize sodium intakes in the adult population. Further refinement of the *SALT* may be required to address issues identified that could improve the congruence to 24-h recalls or dietary records.

## Supplementary information

**Additional file 1.** Sodium AnaLysis Tool (*SALT*)

## Data Availability

The datasets used and/or analysed during the current study are available from the corresponding author on reasonable request.

## Abbreviations

UL: Tolerable Upper Intake Level
SALT: Sodium AnaLysis Tool
SD: Standard Deviation
IOM: Institute of Medicine
CCHS: Canadian Community Health Survey
US: United States
NHANES: National Health and Nutrition Examination Survey
FFQ: Food Frequency questionnaire
DHQ: Diet History Questionnaire
CNF: Canadian Nutrient File
GNPD: Global New Products Database
PHAC: Public Health Agency of Canada
REB: Research Ethics Board
SIDE: Software for Intake Distribution
SPSS: Statistical Statistical Package for the Social Sciences
B-A: Bland-Altman
K: Kappa
m24HR: Mean of three, 24-h dietary recalls
