# Supplementary material for: Development and validation of a sodium AnaLysis tool (SALT)
Source: Nutr J. 2020 Jun 15;19:55. doi: 10.1186/s12937-020-00555-7 (PMC7296949; doi:10.1186/s12937-020-00555-7)
Supplement: Supplementary file 1 — Additional file 1. Sodium AnaLysis Tool (SALT) [file 12937_2020_555_MOESM1_ESM.pdf]

Participant Code:____________________ **Sodium AnaLysis Tool (SALT)** Date:______________

	How often in the past 4 weeks									
**EXAMPLE 1:** If you **usually** ate breads, croissants, bagels, rolls, biscuits or wraps **once** everyday, you'll write this:	Unsalted, low or reduced sodium	Never or less than once a month	1-3 per month	4-6 per month	1-2 per week	3-4 per week	5-6 per week	1 per day	2-3 per day	4+ per day
**Bread, croissants, bagels, rolls, biscuits and flatbreads** including hot dog and hamburger buns, tortillas								x		

	How often in the past 4 weeks									
**EXAMPLE 2:** If you **usually** ate reduced sodium breads, croissants, bagels, rolls, biscuits or wraps **once** everyday, you'll write this:	Unsalted, low or reduced sodium	Never or less than once a month	1-3 per month	4-6 per month	1-2 per week	3-4 per week	5-6 per week	1 per day	2-3 per day	4+ per day
**Bread, croissants, bagels, rolls, biscuits and flatbreads** including hot dog and hamburger buns, tortillas	x							x		

	How often in the past 4 weeks									
	Unsalted, low or reduced sodium	Never or less than once a month	1-3 per month	4-6 per month	1-2 per week	3-4 per week	5-6 per week	1 per day	2-3 per day	4+ per day
**Bread, croissants, bagels, rolls, biscuits and flatbreads** including hot dog and hamburger buns, tortillas										
**Baked goods** such as cakes, muffins, loaves, pie, donuts, pastries, sweet rolls										
**Cookies and granola bars** including cereal bars and marshmallow rice squares										
**Breakfast cereals** including oatmeal, granola, muesli										
**Pancakes, crepes, waffles and french toast**										
**Butter and margarine**										
**Frozen yogourt, ice cream, pudding and custard**										
**Yogourt** including yogourt beverages										
**Milk products and substitutes *(not in tea/coffee)*** including chocolate/flavoured milk, rice/almond/soy beverage, milk shakes										
**Cheese *(not in mixed dishes)*** such as cheddar, swiss, brie, parmesan, processed cheese slice or spread, cream cheese, cottage cheese										
**Wieners, sausages and substitutes**										
**Processed meat and substitutes** such as bacon, luncheon meat, pates and spreads, pepperoni, jerky										
**French fries and deep fried vegetables** including hashbrowns, onion rings, zucchini sticks, vegetable tempura										
**Side salads *(excluding green salads)*** such as pasta salad, potato salad, coleslaw, bean salad										
**Vegetables and legumes *(canned)***										
**Vegetables and legumes *(raw or cooked, plain or fat added)*** including green salads and mashed potatoes										
**Vegetable and legume dishes** such as dahl, chickpea curry, broccoli with cheese sauce, creamed peas, scalloped potatoes, vegetable stew or stir-fry										
**Salad dressings** including mayonnaise										
**Gravies**										
**Condiments** such as soy sauce, ketchup, relish, tartar sauce										

Participant Code:____________________ **Sodium AnaLysis Tool (SALT)** Date:______________

Questionnaire Continued…….	Unsalted, low or reduced sodium	Never or less than once a month	1-3 per month	4-6 per month	1-2 per week	3-4 per week	5-6 per week	1 per day	2-3 per day	4+ per day
	How often in the past 4 weeks									
**Pickled foods** such as dill pickles, pickled vegetables, sauerkraut, olives, pickled eggs										
**Pizza**										
**Stuffed pastry** such as samosa, meat pies, spanakopita, meat filled bun, sausage roll, dumplings (dim sum)										
**Mexican dishes** such as burrito, chimichanga, enchilada, taco salad, nacho, quesadilla, fajita										
**Soups *(canned or prepared from dehydrated mix)***										
**Soups *(homemade or restaurant)***										
**Baked beans and chili *(canned)***										
**Baked beans and chili *(homemade or restaurant)***										
**Pasta, rice and other grains *(plain or fat added)*** such as pasta noodles, rice noodles, white or brown rice, quinoa, couscous										
**Pasta *(canned or prepared from dry mix)*** such as macraoni and cheese fettucini alfredo, pasta carbonara, hamburger and noodles with sauce, ravioli										
**Pasta and noodle dishes *(homemade, frozen or restaurant)*** such as spaghetti, macaroni and cheese, lasagna, ravioli, pad thai, lo mein										
**Rice dishes** such as fried rice, rice pilaf, risotto, sushi										
**Eggs *(plain or fat added)*** such as poached, fried, scrambled eggs										
**Egg dishes** such as omelettes, egg salad, quiche, eggs benedict										
**Meat, fish and shellfish, poultry *(canned)*** such as tuna, crab, salmon, ham, corned beef, chicken										
**Meat, fish and shellfish, poultry and substitutes *(plain or fat added)*** such as pork chop, steak, chicken breast, hamburger patty, fried tofu										
**Meat, fish and shellfish, poultry and substitutes *(mixed dishes)*** such as beef with vegetables, chicken parmigiana, sweet and sour pork, tofu dishes										
**Nuts and seeds** such as trail mix, almonds, peanut butter, sunflower seeds										
**Snack foods** such as chips, crackers, popcorn, pretzel, rice cakes										
**Dips** such as salsa, hummus, french onion, spinach dip, tzaziki										

**How often do you usually use no salt added, low or reduced sodium products when you cook, such as low-sodium broth or no salt added canned tomatoes?**									
**How often do you add salt in cooking?**									
**How often do you add salt to your food at the table?**									
